# National, Regional, State, and Selected Local Area Vaccination Coverage Among Adolescents Aged 13–17 Years — United States, 2018

**DOI:** 10.15585/mmwr.mm6833a2

**Published:** 2019-08-23

**Authors:** Tanja Y. Walker, Laurie D. Elam-Evans, David Yankey, Lauri E. Markowitz, Charnetta L. Williams, Benjamin Fredua, James A. Singleton, Shannon Stokley

**Affiliations:** ^1^Immunization Services Division, National Center for Immunization and Respiratory Diseases, CDC; ^2^Division of Viral Diseases, National Center for Immunization and Respiratory Diseases, CDC; ^3^Leidos Health, Inc., Atlanta, Georgia.

The Advisory Committee on Immunization Practices (ACIP) recommends routine vaccination of persons aged 11–12 years to protect against certain diseases, including human papillomavirus (HPV)–associated cancers, meningococcal disease, and pertussis (*1*). A booster dose of quadrivalent meningococcal conjugate vaccine (MenACWY) is recommended at age 16 years, and serogroup B meningococcal vaccine (MenB) may be administered to persons aged 16–23 years (*1*). To estimate vaccination coverage among adolescents in the United States, CDC analyzed data from the 2018 National Immunization Survey–Teen (NIS-Teen) which included 18,700 adolescents aged 13–17 years.* During 2017–2018, coverage with ≥1 dose of HPV vaccine increased from 65.5% to 68.1%, and the percentage of adolescents up-to-date^†^ with the HPV vaccine series increased from 48.6% to 51.1%, although the increases were only observed among males. Vaccination coverage increases were also observed for ≥1 MenACWY dose (from 85.1% to 86.6%) and ≥2 MenACWY doses (from 44.3% to 50.8%). Coverage with tetanus and reduced diphtheria toxoids and acellular pertussis vaccine (Tdap) remained stable at 89%. Disparities in coverage by metropolitan statistical area (MSA)^§^ and health insurance status identified in previous years persisted (*2*). Coverage with ≥1 dose of HPV vaccine was higher among adolescents whose parents reported receiving a provider recommendation; however, prevalence of parents reporting receiving a recommendation for adolescent HPV vaccination varied by state (range = 60%–91%). Supporting providers to give strong recommendations and effectively address parental concerns remains a priority, especially in states and rural areas where provider recommendations were less commonly reported.

NIS-Teen is an annual survey that monitors vaccines received by adolescents aged 13–17 years in the 50 states, the District of Columbia, selected local areas, and U.S. territories.^¶^ NIS-Teen is conducted among parents and guardians of eligible adolescents identified using a random-digit–dialed sample of cell phone numbers.** During the telephone interview, information is obtained on the sociodemographic characteristics of the teen and household, and contact information and consent to contact the teen’s vaccination providers are requested. Vaccination providers identified during the interview are mailed a questionnaire requesting the vaccination history from the teen’s medical record.^††^ Vaccination coverage estimates are based on provider-reported vaccination histories. This report presents vaccination coverage estimates for 18,700 adolescents (8,928 [48%] females and 9,772 [52%] males) aged 13–17 years with adequate provider data.^§§^ The overall Council of American Survey Research Organizations response rate was 23.3%, and only 48.3% of adolescents with completed interviews had adequate provider data.

Previously described NIS-Teen methodology, including methods for weighting and synthesizing provider-reported vaccination histories (https://www.cdc.gov/vaccines/imz-managers/nis/downloads/NIS-TEEN-PUF17-DUG.pdf) was used. Beginning in 2018, NIS-Teen used a single-frame sample of cell phone lines. The landline telephone–sample frame that was used from 2006 through 2017 was dropped because of the declining number of landline-only households in the United States (https://www.cdc.gov/vaccines/imz-managers/coverage/teenvaxview/pubs-presentations/dual-to-single-frame-teen.html). Data were weighted and analyzed to account for the complex sampling design. T-tests were used to assess vaccination coverage differences by survey year (2018 compared with 2017) and between demographic subgroups. P-values <0.05 were considered statistically significant. SAS-callable SUDAAN (version 11; SAS Institute) was used to conduct all analyses.

## National Vaccination Coverage

In 2018, 51.1% of adolescents aged 13–17 years were up to date with the HPV vaccine series, and 68.1% had received ≥1 dose of HPV vaccine (Table 1) (Figure). During 2017–2018, the increase in HPV vaccination coverage was attributable to increases among males only (increase of 4.4 percentage points in males who were up to date versus 0.6 in females). Coverage with ≥1 MenACWY dose increased by 1.5 percentage points to 86.6%. Among persons aged 17 years, coverage with ≥2 MenACWY doses increased by 6.5 percentage points to 50.8%. Coverage with ≥1 dose of MenB among persons aged 17 years was 17.2% (95% confidence interval = 14.9%–19.9%). No significant increases were observed for coverage with ≥3 hepatitis B doses; ≥2 measles, mumps, and rubella vaccine doses; and ≥1 and ≥2 varicella vaccine doses among adolescents without a history of varicella disease (Table 1).

**TABLE 1:** Estimated coverage with selected vaccines and doses among adolescents aged 13–17* years, by age at interview — National Immunization Survey–Teen (NIS-Teen), United States, 2018

Vaccine	Age at interview (yrs), % (95% CI)^†^					Total	
	13	14	15	16	17	2018	2017
	(n = 3,852)	(n = 3,875)	(n = 3,741)	(n = 3,751)	(n = 3,481)	(n = 18,700)	(n = 20,949)
**Tdap^§^ ≥1 dose**	87.1 (85.0–89.0)	87.7 (85.4–89.7)	89.7 (87.8–91.4)	89.0 (87.1–90.6)	91.0 (89.5–92.4)^¶^	88.9 (88.0–89.7)	88.7 (87.8–89.6)
MenACWY**							
≥1 dose	86.3 (84.2–88.1)	86.2 (84.0–88.1)	86.1 (83.7–88.2)	86.3 (84.0–88.3)	88.1 (86.3–89.6)	86.6 (85.6–87.5)^††^	85.1 (84.2–86.1)
≥2 doses**^§§^**	NA	NA	NA	NA	50.8 (47.7–53.8)	50.8 (47.7–53.8)^††^	44.3 (41.4–47.2)
**HPV^¶¶^ vaccine**							
All adolescents							
UTD***	39.9 (37.0–42.9)	50.3 (47.3–53.2)^¶^	54.0 (51.0–56.9)^¶^	54.5 (51.5–57.5)^¶^	57.5 (54.4–60.5)^¶^	51.1 (49.8–52.5)^††^	48.6 (47.3–49.9)
≥1 dose	62.6 (59.7–65.4)	66.9 (64.1–69.6)^¶^	69.7 (66.9–72.3)^¶^	71.2 (68.5–73.8)^¶^	70.1 (67.3–72.8)^¶^	68.1 (66.8–69.3)^††^	65.5 (64.3–66.7)
Females							
UTD	38.9 (35.0–42.9)	52.7 (48.5–56.8)^¶^	54.7 (50.4–59.0)^¶^	57.5 (53.3–61.6)^¶^	66.0 (61.8–70.1)^¶^	53.7 (51.8–55.6)	53.1 (51.2–55.0)
≥1 dose	61.1 (56.9–65.2)	68.6 (64.4–72.5)^¶^	70.7 (66.5–74.5)^¶^	73.5 (69.8–76.8)^¶^	76.3 (72.2–80.0)^¶^	69.9 (68.1–71.6)	68.6 (66.9–70.2)
Males							
UTD	40.9 (36.5–45.3)	47.7 (43.6–51.8)^¶^	53.2 (49.1–57.3)^¶^	51.8 (47.5–56.1)^¶^	50.0 (45.7–54.3)^¶^	48.7 (46.8–50.6)^††^	44.3 (42.6–46.0)
≥1 dose	64.0 (59.9–67.9)	65.1 (61.3–68.7)	68.7 (65.0–72.1)	69.2 (65.2–73.0)	64.7 (60.7–68.5)	66.3 (64.6–68.0)^††^	62.6 (60.9–64.2)
**MenB ≥1 dose^†††^**	NA	NA	NA	NA	17.2 (14.9–19.9)	17.2 (14.9–19.9)	14.5 (12.3–17.1)
**MMR ≥2 doses**	93.5 (92.1–94.7)	93.0 (91.6–94.2)	91.8 (89.9–93.3)	90.5 (88.4–92.2)^¶^	90.9 (89.2–92.4)^¶^	91.9 (91.2–92.6)	92.1 (91.3–92.8)
**Hepatitis B vaccine ≥3 doses**	93.1 (91.5–94.5)	93.0 (91.5–94.3)	91.6 (89.1–93.5)	91.1 (89.3–92.6)	91.8 (90.1–93.2)	92.1 (91.3–92.8)	91.9 (91.1–92.6)
**Varicella vaccine**							
History of varicella disease^§§§^	9.8 (8.1–11.9)	10.3 (8.5–12.4)	11.8 (10.0–13.9)	12.4 (10.7–14.3)	15.0 (13.2–17.1)^¶^	11.9 (11.0–12.7)^††^	13.2 (12.3–14.2)
No history of varicella disease							
≥1 dose vaccine	95.4 (94.2–96.5)	95.4 (94.2–96.3)	94.1 (92.1–95.6)	94.3 (92.7–95.5)	95.2 (93.9–96.3)	94.9 (94.3–95.4)	95.5 (94.8–96.1)
≥2 doses vaccine	92.1 (90.5–93.4)	91.3 (89.6–92.8)	89.8 (87.4–91.8)	86.6 (84.3–88.7)^¶^	87.9 (85.4–90.1)^¶^	89.6 (88.7–90.4)	88.6 (87.6–89.5)
History of varicella or ≥2 vaccine doses	92.9 (91.4–94.1)	92.2 (90.6–93.5)	91.0 (88.9–92.7)	88.3 (86.2–90.1)^¶^	89.7 (87.5–91.6)^¶^	90.8 (90.0–91.6)	90.1 (89.3–90.9)

**Abbreviations:** CI = confidence interval; HPV = human papillomavirus; MenACWY = quadrivalent meningococcal conjugate vaccine; MenB = serogroup B meningococcal vaccine; MMR = measles, mumps, and rubella vaccine; NA = not applicable; Tdap = tetanus toxoid, reduced diphtheria toxoid, and acellular pertussis vaccine; UTD = up-to-date.

* Adolescents (N = 18,700) in the 2018 NIS-Teen were born January 2000–February 2006.

^†^ Estimates with 95% CIs >20 might be unreliable.

^§^ Includes percentages receiving Tdap vaccine at age ≥10 years.

^¶^ Statistically significant difference (p<0.05) in estimated vaccination coverage by age; reference group was adolescents aged 13 years.

** Includes percentages receiving MenACWY or meningococcal-unknown type vaccine.

^††^ Statistically significant difference (p<0.05) compared with 2017 NIS-Teen estimates.

^§§^ ≥2 doses of MenACWY or meningococcal-unknown type vaccine. Calculated only among adolescents who were aged 17 years at interview. Does not include adolescents who received 1 dose of MenACWY vaccine at age ≥16 years.

^¶¶^ HPV vaccine, 9-valent (9vHPV), quadrivalent (4vHPV), or bivalent (2vHPV). Percentages are reported among females and males combined (N = 18,700) and for females only (N = 8,928) and males only (N = 9,772).

*** HPV UTD includes those with ≥3 doses, and those with 2 doses when the first HPV vaccine dose was initiated at age <15 years, and there was at least 5 months minus 4 days between the first and second dose. This update to the HPV recommendation occurred in December 2016 (https://www.cdc.gov/mmwr/volumes/65/wr/mm6549a5.htm).

^†††^ ≥1 dose of MenB. Calculated only among adolescents aged 17 years at interview. Administered based on individual clinical decision.

^§§§^ By parent/guardian report or provider records.

**FIGURE:**
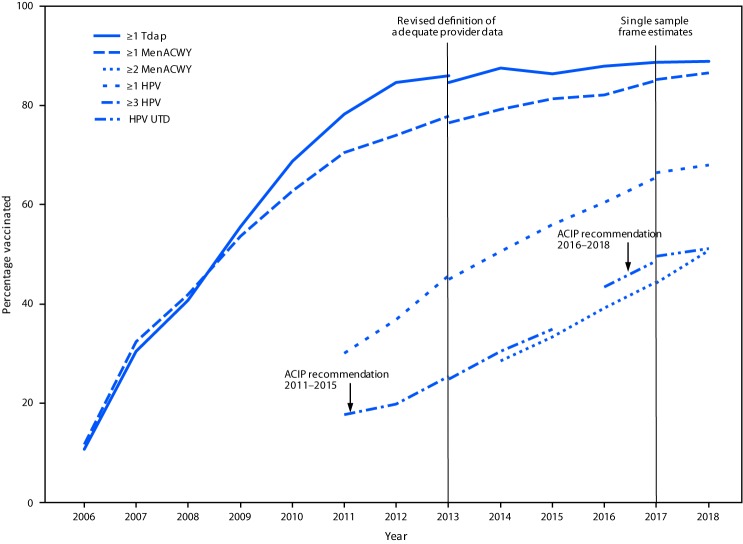
Estimated vaccination coverage with selected vaccines and doses* among adolescents aged 13–17 years, by survey year and Advisory Committee on Immunization Practices (ACIP) recommendations^†^ — National Immunization Survey–Teen (NIS-Teen),^§^^,^^¶^ United States, 2006–2018 **Abbreviations:** HPV = human papillomavirus vaccine; MenACWY = quadrivalent meningococcal conjugate vaccine; Tdap = tetanus toxoid, reduced diphtheria toxoid, and acellular pertussis vaccine; UTD = up-to-date. * ≥1 dose Tdap at or after age 10 years; ≥1 dose MenACWY or meningococcal-unknown type vaccine; ≥2 doses MenACWY or meningococcal-unknown type vaccine, calculated only among adolescents aged 17 years at time of interview. Does not include adolescents who received their first and only dose of MenACWY at or after age 16 years; HPV vaccine, nine-valent (9vHPV), quadrivalent (4vHPV), or bivalent (2vHPV). HPV UTD includes those with ≥3 doses and those with 2 doses when the first HPV vaccine dose was initiated before age 15 years and at least 5 months minus 4 days elapsed between the first and second dose. ^†^ ACIP revised the recommended HPV vaccination schedule in late 2016. The recommendation changed from a 3-dose to 2-dose series with appropriate spacing between receipt of the first and second dose for immunocompetent adolescents initiating the series before the 15th birthday. Three doses are still recommended for adolescents initiating the series between the ages of 15 and 26 years. Because of the change in recommendation, the graph includes estimates for ≥3 doses HPV from 2011 to 2015 and the HPV UTD estimate from 2016 to 2018. The routine ACIP recommendation for HPV vaccination was made for females in 2006 and for males in 2011. Because HPV vaccination was not recommended for males until 2011, coverage for all adolescents was not measured before that year. ^§^ NIS-Teen implemented a revised adequate provider data definition (APD) in 2014 and retrospectively applied the revised APD definition to 2013 data. Estimates using different APD definitions might not be directly comparable. ^¶^ NIS-Teen moved from a dual landline and cell phone sampling frame to a single cell phone sample frame in 2018, and estimates using 2017 data were calculated two ways, using the dual frame and retrospectively using the single cell phone sampling frame.

## Vaccination Coverage by Selected Characteristics

Coverage for all measures of HPV and MenACWY vaccination and ≥2 varicella vaccine doses among adolescents without a history of varicella disease were lower among adolescents living in non-MSA areas than in those living in MSA principal cities (Table 2). The largest differences were in HPV up-to-date status (15.4 percentage point difference) and ≥2-dose MenACWY coverage (19.7 percentage point difference). Coverage differences between adolescents living in MSA nonprincipal cities and MSA principal cities were observed for HPV vaccination measures (5.3 and 7.0 percentage point differences for receipt of ≥1 dose and being up-to-date, respectively) and ≥3 hepatitis B doses (1.7 percentage points). Compared with adolescents with private health insurance, those with Medicaid had higher HPV vaccination coverage (8.8 and 5.5 percentage points higher for receipt of ≥1 dose and being up-to-date, respectively) (Table 2). Uninsured adolescents had lower vaccination coverage, with differences ranging from 4.4 percentage points (≥1 varicella vaccine dose) to 18.7 percentage points (≥2 MenACWY doses) lower than did adolescents with private insurance. Vaccination coverage estimates also differed by race/ethnicity (Supplementary Table 1, https://stacks.cdc.gov/view/cdc/80676); poverty level (Supplementary Table 2, https://stacks.cdc.gov/view/cdc/80677); and jurisdiction (Supplementary Table 3, https://stacks.cdc.gov/view/cdc/80678). During 2014–2018, ≥1dose-HPV vaccination coverage increased an average of 4.4 percentage points per year nationally. (Supplementary Table 4, https://stacks.cdc.gov/view/cdc/80679).

**TABLE 2:** Estimated vaccination coverage with selected vaccines and doses among adolescents* aged 13–17 years by metropolitan statistical area^†^ and health insurance status^§^ — National Immunization Survey–Teen (NIS-Teen), United States, 2018

Vaccine	MSA% (95% CI)^¶^			Health insurance status % (95% CI)^¶^			
	Non-MSA	MSA nonprincipal city	MSA principal city	Private insurance only	Any Medicaid	Other insurance	Uninsured
	(n = 3,593)	(n = 7,543)	(n = 7,564)	(n = 10,404)	(n = 5,999)	(n = 1,516)	(n = 781)
Tdap** ≥1 dose	86.8 (84.8–88.5)	89.7 (88.4–90.8)	88.6 (87.1–89.9)	90.1 (89.0–91.2)	88.2 (86.6–89.6)**^††^**	85.6 (82.3–88.3)**^††^**	85.1 (80.7–88.6)**^††^**
**MenACWY^§§^**							
≥1 dose	79.5 (77.3–81.6)**^††^**	88.3 (87.1–89.4)	86.5 (84.7–88.0)	87.6 (86.4–88.8)	86.5 (84.8–88.0)	84.3 (81.1–87.0)**^††^**	78.3 (72.7–83.0)**^††^**
≥2 doses^¶¶^	34.6 (28.5–41.2)**^††^**	51.5 (46.7–56.2)	54.3 (49.7–58.9)	52.8 (48.6–56.9)	52.4 (46.9–57.8)	38.6 (30.0–48.0)**^††^**	34.1 (21.6–49.4)**^††^**
HPV*** vaccine							
UTD^†††^	40.7 (38.1–43.5)**^††^**	49.1 (47.1–51.0)**^††^**	56.1 (53.9–58.3)	50.2 (48.4–52.0)	55.7 (53.4–58.1)**^††^**	45.1 (40.9–49.3)**^††^**	35.5 (30.1–41.4)**^††^**
≥1 dose	59.5 (56.8–62.2)**^††^**	66.6 (64.8–68.4)**^††^**	71.9 (69.8–73.9)	65.6 (63.8–67.3)	74.4 (72.3–76.3)**^††^**	62.6 (58.5–66.5)	56.2 (50.1–62.2)**^††^**
**MMR ≥2 doses**	90.1 (88.1–91.8)	92.3 (91.2–93.2)	92.0 (90.8–93.1)	92.8 (91.9–93.6)	92.0 (90.6–93.1)	90.1 (87.3–92.3)**^††^**	84.2 (78.6–88.5)**^††^**
**Hepatitis B ≥3 vaccine doses**	90.7 (88.8–92.4)	93.1 (92.1–94.0)**^††^**	91.4 (89.9–92.6)	93.0 (91.9–93.9)	92.1 (90.8–93.3)	90.5 (87.8–92.6)	84.1 (78.5–88.4)**^††^**
**Varicella vaccine**							
History of varicella^§§§^	15.0 (13.1–17.0)**^††^**	10.6 (9.6–11.8)	12.4 (10.9–14.0)	9.8 (8.8–10.9)	13.4 (11.8–15.1)**^††^**	13.8 (11.1–17.1)**^††^**	20.4 (16.2–25.4)**^††^**
Among adolescents with no history of varicella disease							
≥1 varicella vaccine dose	93.4 (91.5–94.9)	95.0 (94.1–95.8)	95.1 (94.0–96.0)	95.7 (94.9–96.3)	94.4 (93.2–95.4)	93.3 (90.7–95.1)**^††^**	91.3 (86.0–94.7)**^††^**
≥2 varicella vaccine doses	86.4 (84.1–88.4)**^††^**	89.8 (88.3–91.1)	90.2 (88.8–91.4)	90.5 (89.3–91.7)	89.4 (87.8–90.8)	86.7 (83.4–89.4)**^††^**	83.8 (77.6–88.5)**^††^**
History of varicella or ≥2 vaccine doses	88.5 (86.5–90.2)**^††^**	90.9 (89.6–92.0)	91.4 (90.1–92.5)	91.5 (90.3–92.5)	90.8 (89.4–92.1)	88.5 (85.6–90.9)**^††^**	87.1 (82.0–90.9)

**Abbreviations:** CI = confidence interval; HPV = human papillomavirus; MenACWY = quadrivalent meningococcal conjugate vaccine; MMR = measles, mumps, and rubella vaccine; MSA= metropolitan statistical area; Tdap = tetanus toxoid, reduced diphtheria toxoid, and acellular pertussis vaccine; UTD = up-to-date.

* Adolescents (N = 18,700) in the 2018 NIS-Teen were born January 2000–February 2006.

^†^ MSA status was determined based on household-reported county of residence, and was grouped into three categories: MSA principal city, MSA nonprincipal city, and non-MSA. MSA and principal city were as defined by the U.S. Census Bureau (https://www.census.gov/programs-surveys/metro-micro.html). Non-MSA areas include urban populations not located within an MSA as well as completely rural areas.

^§^ Adolescents' health insurance status was reported by parent or guardian. “Other insurance” includes the Children’s Health Insurance Program, military insurance, Indian Health Service, and any other type of health insurance not mentioned elsewhere.

^¶^ Estimates with CIs >20 might be unreliable.

** Includes percentages receiving Tdap vaccine at age ≥10 years.

^††^ Statistically significant difference (p<0.05) in estimated vaccination coverage by MSA or health insurance status. The referent groups were adolescents living in MSA principal city areas and adolescents with private insurance only, respectively.

^§§^ Includes percentages receiving MenACWY and meningococcal-unknown type vaccine.

^¶¶^ ≥2 doses of MenACWY or meningococcal-unknown type vaccine. Calculated only among adolescents aged 17 years at interview. Does not include adolescents who received 1 dose of MenACWY vaccine at age ≥16 years.

*** HPV vaccine, nine-valent (9vHPV), quadrivalent (4vHPV), or bivalent (2vHPV) in females and males combined.

^†††^ HPV UTD includes those with ≥3 doses, and those with 2 doses when the first HPV vaccine dose was initiated at age <15 years, and there was at least 5 months minus 4 days between the first and second dose. This update to the HPV recommendation occurred in December 2016 (https://www.cdc.gov/mmwr/volumes/65/wr/mm6549a5.htm).

^§§§^ By parent/guardian report or provider records.

## Provider Recommendation for HPV Vaccination

Overall, 77.5% of parents reported receiving a provider recommendation for adolescent HPV vaccination; prevalence varied by state, ranging from 59.5% in Mississippi to 90.7% in Massachusetts (Supplementary Figure, https://stacks.cdc.gov/view/cdc/80682) (Supplementary Table 5, https://stacks.cdc.gov/view/cdc/80680). Nationally, ≥1-dose HPV vaccination coverage was higher among adolescents whose parents reported receiving a provider recommendation (74.7%) than among those whose parents reported not receiving a provider recommendation (46.7%) (Supplementary Table 5, https://stacks.cdc.gov/view/cdc/80680). Fewer parents living in non-MSA areas reported receiving a provider recommendation than did those living in MSA principal cities (70.3% versus 77.4%) (Supplementary Table 6, https://stacks.cdc.gov/view/cdc/80681).

## Discussion

In 2018, U.S. adolescent vaccination coverage with ≥1 and ≥2 doses of MenACWY, ≥1 dose of HPV vaccine and being up-to-date with HPV vaccination continued to improve. Coverage with ≥1 Tdap dose remains high but appears to have stabilized. Although HPV vaccination coverage improved, increases among all adolescents were modest compared with increases in previous years and were observed only among males. Since 2011,^¶¶^ coverage has increased gradually among females and more rapidly among males. However, only approximately half of adolescents have been fully vaccinated for HPV.

HPV vaccination coverage was higher among adolescents whose parent reported receiving a provider recommendation. Thus, the provider recommendation continues to be a strong predictor of HPV vaccination (*3*,*4*). However, even when a provider recommendation was given, only 75% accepted the vaccine, suggesting that there are other reasons adolescents are not being vaccinated. Equipping providers with the tools they need to give strong recommendations that emphasize the importance of HPV vaccination in preventing cancer and effectively address parental concerns is a priority, especially in states where provider recommendations were less commonly reported. Resources on the importance of HPV vaccination and videos demonstrating how to give a recommendation are available to facilitate discussion between providers, teens, and their parents (https://www.cdc.gov/vaccines/vpd/hpv/hcp/resources.html).

Coverage disparities persisted for some vaccines by MSA status. The disparity in HPV vaccination coverage by MSA status is not well understood; however, the lower prevalence of provider recommendations in non-MSA areas might be a factor. In one study, parents and guardians in the rural South indicated that they did not have enough information on the vaccine or its purpose (*5*). Efforts to ensure that rural health care providers have the resources and training necessary to educate parents and guardians about the benefits of HPV vaccination as a cancer prevention tool might increase the number of adolescents protected against diseases caused by HPV.

Vaccination coverage was significantly lower among uninsured adolescents than among those with private insurance. Adolescents without health insurance are eligible to receive vaccines through the Vaccines for Children (VFC) program.*** Lack of parental awareness of (*6*) and misconceptions about the program, including that it is only for infants and younger children, might serve as barriers (*7*). Increasing parental awareness and knowledge of the VFC program should improve vaccination coverage among uninsured adolescents. Providers can assist by ensuring that their health care practice routinely screen patients for eligibility and counsel families about the VFC program.

The findings in this report are subject to at least seven limitations. First, the overall Council of American Survey Research Organizations response rate was low, and fewer than half of adolescents with completed interviews had adequate provider data. Second, bias in estimates might remain even after adjustment for household and provider nonresponse and landline-only and phoneless households.^†††^ Third, changes in estimates of vaccination coverage from 2017 to 2018 should be interpreted with caution, given the transition from dual landline- and cellular- to single-cellular telephone-sampling frame in 2018. Fourth, estimates stratified by jurisdiction might be unreliable because of small sample sizes. Fifth, multiple statistical tests were conducted, and a small number might be significant because of chance alone. Sixth, coverage with ≥2 doses of MenACWY and ≥1 dose of MenB might be underestimated because MenB and second MenACWY dose may be administered at age >17 years (*1*), and NIS-Teen includes adolescents aged 13–17 years. Finally, the “provider recommendation” variable is based on parental report and thus subject to recall bias.

It is encouraging that HPV vaccination coverage among boys continues to increase; however, the lack of an increase among girls is concerning. In the United States, an estimated 34,800 cases of cancer caused by HPV occur each year; 32,100 (92%), including 59% among women, would be preventable by the 9-valent HPV vaccine (*8*). Although, HPV vaccination has resulted in large declines in the prevalence of vaccine type HPV infections among adolescent girls and young adults (*9*), as well as decreases in cervical precancers (*10*), continuing to improve HPV vaccination coverage for all adolescents, male and female, will ensure they are protected from HPV infection and diseases caused by HPV, including cancers.

**Summary**

**What is already known about this topic?**

Vaccines are recommended for adolescents to prevent diphtheria, pertussis, tetanus, meningococcal disease, and cancers caused by human papillomavirus (HPV).

**What is added by this report?**

In 2018, adolescent vaccination coverage in the United States continued to improve for meningococcal and HPV vaccines (primarily from increases among boys) and remains high for tetanus and reduced diphtheria toxoids and acellular pertussis vaccine. Adolescents whose parents reported having received a provider recommendation were more likely to have received HPV vaccination compared with adolescents whose parents did not report a provider recommendation.

**What are the implications for public health care?**

Providing parents and guardians with information and strong, high-quality recommendations are valuable tools for improving HPV vaccination and preventing HPV infection and diseases caused by HPV, including cancers.

## Footnotes

* Eligible participants were born during January 2000–February 2006. Tdap represents coverage with ≥1 Tdap dose at age ≥10 years. MenACWY represents coverage with the quadrivalent meningococcal conjugate vaccine or meningococcal-unknown type vaccine. ACIP published Category B recommendations for the use of serogroup B meningococcal vaccine (MenB) in October 2015 (https://www.cdc.gov/mmwr/preview/mmwrhtml/mm6441a3.htm), with administration preferred at ages 16–18 years. HPV vaccination coverage represents receipt of any HPV vaccine and does not distinguish among 9-valent (9vHPV), quadrivalent (4vHPV), or bivalent (2vHPV) vaccines. Some adolescents might have received more than the 2 or 3 recommended HPV vaccine doses. Except as noted, coverage estimates for ≥1 and ≥2 varicella vaccine doses were obtained among adolescents with no history of varicella disease. Influenza vaccination coverage data are not included in this report but are available online at https://www.cdc.gov/flu/fluvaxview/index.htm.

† Adolescents were considered to be up to date with HPV vaccination if they had received ≥3 doses, or if all of the following applied: 1) they had received 2 doses; 2) the first dose was received before their 15th birthday; and 3) the difference between dates of first and second doses was ≥5 months minus 4 days, the absolute minimum interval between the first and second doses (https://www.cdc.gov/vaccines/programs/iis/cdsi.html).

§ MSA status was determined based on household reported city and county of residence, and was grouped into three categories: MSA principal city, MSA nonprincipal city, and non-MSA. MSA and principal city were as defined by the U.S. Census Bureau (https://www.census.gov/programs-surveys/metro-micro.html). Non-MSA areas include urban populations not located within an MSA as well as completely rural areas.

¶ Local areas that received federal Section 317 immunization funds were sampled separately: Chicago, Illinois; New York, New York; Philadelphia County, Pennsylvania; Bexar County, Texas; and Houston, Texas. Two local areas were oversampled: Hidalgo County, Texas and Tarrant County, Texas. Only one territory, Guam, was included as an estimation area in 2018.

** All identified cellular-telephone households were eligible for interview. Sampling weights were adjusted for single-frame (cellular telephone), nonresponse, and noncoverage. A description of NIS-Teen single-frame survey methodology and its effect on reported vaccination estimates is available at https://www.cdc.gov/vaccines/imz-managers/coverage/teenvaxview/pubs-presentations/dual-to-single-frame-teen.html.

†† For the telephone samples for the states and local areas, the overall Council of American Survey Research Organizations (CASRO) response rate was 23.3%. For adolescents with completed interviews, 48.3% had adequate provider data. For Guam, the CASRO response rate was 22.4%, and 55.0% of adolescents with completed interviews had adequate provider data. In 2017, among completed interviews with adequate provider data, 17% (3,572) were from the landline sample, and 83% (17,377) were from the cell phone sample. The CASRO response rate is the product of three other rates: 1) the resolution rate (the proportion of telephone numbers that can be identified as either for business or residence); 2) the screening rate (the proportion of qualified households that complete the screening process); and 3) the cooperation rate (the proportion of contacted eligible households for which a completed interview is obtained).

§§ Adolescents from Guam (309) were excluded from the national estimates.

¶¶ ACIP recommended a 3-dose series of HPV vaccine for girls aged 11 to 12 years in 2006 (https://www.cdc.gov/Mmwr/Preview/Mmwrhtml/rr5602a1.htm) and for boys in 2011 (https://www.cdc.gov/mmwr/preview/mmwrhtml/mm6050a3.htm). In 2016, the recommendation was revised to a 2-dose series for immunocompetent adolescents beginning the series before their 15th birthday with appropriate spacing between doses. All other persons are recommended to complete a 3 dose vaccine series (https://www.cdc.gov/mmwr/volumes/65/wr/mm6549a5.htm).

*** Children and adolescents aged ≤18 years who are Medicaid-eligible, uninsured, or American Indian/Alaska Native (as defined by the Indian Health Care Improvement Act) are eligible to receive vaccines from providers through the VFC program. Children and adolescents categorized as “underinsured” (because their health plans do not include coverage for recommended vaccinations) are eligible to receive VFC vaccines if they are served by a rural health clinic or federally qualified health center or under an approved deputization agreement. (https://www.cdc.gov/vaccines/programs/vfc/providers/eligibility.html)

††† In a sensitivity analysis of 2013 estimates using comparisons to vaccination data collected from a sample of National Health Interview Survey (NHIS), respondents indicated that estimated coverage with ≥1 Tdap dose, ≥1 MenACWY dose, and ≥1 HPV dose (females) were within two percentage points of true estimates (https://www.cdc.gov/vaccines/imz-managers/nis/downloads/NIS-TEEN-PUF17-DUG.pdf). These differences were within the margin of plausible error of the model. The model accounted for three types of error: incomplete sample frame (e.g., exclusion of teens in households with no type of telephone service); nonresponse bias; and incomplete ascertainment of vaccination status by NIS-Teen provider record check.
